# From mannequins to humans – are manual therapy motor skills transferable? A mixed-methods study

**DOI:** 10.1186/s12909-026-08806-7

**Published:** 2026-02-14

**Authors:** Martha Funabashi, Nicole M. Smith, Kitlyn Wong, Angela Gnjatic, Orion SmithBrudenell, David Starmer, Samuel J. Howarth, Grand Choi, Casper Nim

**Affiliations:** 1 Canadian Memorial Chiropractic College, Toronto, Canada; 2 Department of Sport Science and Clinical Biomechanics, University of Southern Denmark, Campusvej 55, Odense M, DK-5230 Denmark; 3 Department of Chiropractic, Université du Québec à Trois-Rivières, Trois- Rivières, Canada; 4 Medical Research Unit, Spine Centre of Southern Denmark, University Hospital of Southern Denmark, Kolding, Denmark; 5 Department of Regional Health Research, University of Southern Denmark, Odense, Denmark

**Keywords:** Spinal manipulative therapy, Manual therapy learning, Simulation-based learning, Motor skill development

## Abstract

**Background:**

Manual therapy, including spinal manipulative therapy (SMT), is a core component of chiropractic education. Simulation tools such as the Human Analogue Mannequin (HAM^®^) have been developed and coupled with force-sensing technology to support safe and structured training of SMT force-time characteristics. However, it remains unclear whether motor skills acquired using these tools are transferable to real-world scenarios.

**Methods:**

This sequential explanatory mixed-methods observational study investigated whether chiropractic students, proficient at applying a set of pre-specified SMT force-time characteristics to the HAM^®^, could replicate those same characteristics in humans. Quantitative data measured SMT force-time characteristics (preload force, peak impulse force, time to peak impulse force) applied on the HAM^®^. Participants meeting the predefined force-time criteria in 4 out of 5 trials proceeded to apply SMTs to a human. Qualitative data from a survey with open-ended questions explored participants’ perceptions of their ability to replicate the SMT and differences between the HAM^®^ and humans.

**Results:**

Ninety-five students participated (56% female, median age: 25 years), and 50 (53%) met the criteria on the HAM^®^. Thirty-seven (74%) of the participants who met the criteria on the HAM^®^ also met the criteria on humans. No significant differences in participant characteristics were found between those who met or did not meet the criteria when applying SMT on humans. Participants who did not meet SMT force-time criteria on either the HAM^®^ or humans often under-applied preload force. Time to peak impulse force criteria was usually met across all trials. Qualitatively, participants perceived that internal factors (e.g., previous experience, emotions, tactile feel) and differences between the HAM^®^ and humans (e.g., stiffness, breathing) influenced their ability to replicate the SMT force-time criteria on humans.

**Conclusion:**

Most students who successfully applied SMT force-time characteristics on a mannequin were able to replicate them on humans. Not meeting the SMT proficiency criteria (particularly in preload force) and the influence of non-biomechanical factors highlights the complexity of manual therapy motor skill development.

**Supplementary Information:**

The online version contains supplementary material available at 10.1186/s12909-026-08806-7.

## Background

Learning and developing the motor skills to apply manual therapy, including spinal manipulative therapy (SMT), is an important part of the curriculum of many healthcare professions, including manual medicine, physiotherapy, chiropractic, osteopathy, among others. Manual therapy is a complex motor skill with varying levels of difficulty depending on the technique used. Like any motor task, learning manual therapy requires pedagogic strategies and structured training sessions based on repetition and feedback [1]. Using humans (fellow students) as receivers during the repetitive practice necessary for developing these skills might be problematic, particularly at the high frequencies (2–3 h per day) usually observed in manual therapy educational programs [2]. This amount of exposure to manual therapy might potentially present risks to students who receive repetitive manual therapy [2]. Minimizing potential harm has been partially responsible for the development of simulation-based training tools designed to enhance learning of manual therapy skills.

Examples of such developed tools include force sensing devices and the human analogue mannequin simulators [3]. Specifically, treatment tables with embedded force plates quantify manual therapy forces and provide trainees with immediate feedback regarding force-time characteristics of manual therapy procedures [4, 5]. It facilitates students’ learning of modulating manual therapy force-time characteristics and several teaching institutions worldwide have integrated force sensing devices into their curriculum to facilitate learning and training. For example, students use information from force sensing devices as feedback to learn to modulate SMT forces to apply low, medium and large force magnitudes with specific thrust durations, mimicking the forces recorded from practicing chiropractors [4, 6, 7].

To complement force sensing devices, high-fidelity human analogue mannequin simulators have been increasingly used in the training and education of healthcare professionals since the 1990s with benefits including enhanced technical skills, communication and decision-making [8]. Specific to manual therapy, participants in previous studies have reported that human analogue mannequins used during their education effectively represent the real-world experience of humans [9, 10]. However, despite its widespread use, the extent to which proficiency of delivering manual therapy to mannequins translates to human receivers remains unclear. Specifically, it remains unknown if students who learn to deliver manual therapies with pre-defined force-time characteristics on mannequins can replicate those same characteristics when performing the same manual therapy on humans. If the ability to apply manual therapy force-time characteristics practiced in mannequins are transferable to when applying it to humans, it would allow for the training of SMT force-time characteristics using a safe and standardized approach. Standardizing SMT force-time training, in turn, may potentially contribute to safer clinical practice by reducing variability and minimizing the risk of excessive force application.

Therefore, this project determined if students who are proficient in applying SMT with predefined parameters of force-time characteristics to mannequins can replicate the same force-time characteristics in humans.

## Methods

### Design

A sequential explanatory mixed-methods observational study design with quantitative priority was used. Quantitative data investigated the proficiency of SMT force-time characteristics, whereas the qualitative data explored participants’ perceptions regarding potential differences and similarities between mannequins and humans. The qualitative data helped to explain the quantitative data by providing context to the observed force-time outcomes.

This study followed a pre-determined protocol that was prospectively uploaded to Open Science Framework (https://osf.io/waks4/). The study is reported following Good Reporting of a Mixed Methods Study (GRAMMS) checklist [11]. This study was approved by the Canadian Memorial Chiropractic College’s (CMCC) Research Ethics Board (REB Approval# 2208B01). This study was conducted in accordance with the ethical standards as laid down in the Declaration of Helsinki. Prior to beginning data collection, all participants reviewed a study information letter and signed an electronic informed consent form.

### Participants and setting

A convenience sample of CMCC students (Toronto, Canada) were invited to participate in the study via email announcements and word of mouth between March 2024 and February 2025. Participants could participate as “providers” (i.e., providing the SMT), as “human receivers” (i.e., receiving the SMT) or both. To be included, participants needed to be at least 18 years old; able to provide a posterior-to-anterior thoracic SMT to a mannequin; and have no current or history of neck, thoracic or lumbar spine pain in the past 3 months. Participants were excluded as “providers” if they presented any injuries or conditions that would prevent them from safely performing or receiving the investigated SMT procedure (e.g., upper extremity pain); and as “receivers” if they had history of spinal surgery or significant pathology (e.g. heart or lung disease, cancer, osteoporosis, spinal infection, neoplasm, or systemic disease).

### Instrumentation

Three-dimensional forces were measured and recorded using the Force Sensing Table Technology (FSTT^®^, Toronto, Ontario, Canada) for all SMT trials. The FSTT^®^ is a validated tool composed of a modified Elite Stationary treatment table (Elite Chiropractic Tables, Jarvis, Ontario, Canada) with an embedded force plate (Advanced Mechanical Technology Inc., Watertown, Massachusetts, USA) [4, 5]. The thoracic portion of the treatment table (with embedded force plate) was mechanically independent from the remainder of the table.

Force data were digitally sampled at a rate of 2000 Hz using a ± 10 V range on a 16-bit analog to digital conversion board. The FSTT^®^ readings were zeroed prior to each SMT application. Previous research has demonstrated excellent construct validity of the FSTT^®^ in quantifying SMT force-time characteristics [5].

The Human Analogue Mannequin (HAM^®^, Toronto, Ontario, Canada) was used as the mannequin simulator. It was developed to simulate a human torso with anthropometrically consistent soft tissue compliance and anatomical landmarks to serve as stand-in for live subjects [12]. Previous studies have used the HAM^®^ in manual therapy investigations and have reported that it represents the real-world experience of humans [9, 10].

**Fig. 1:**
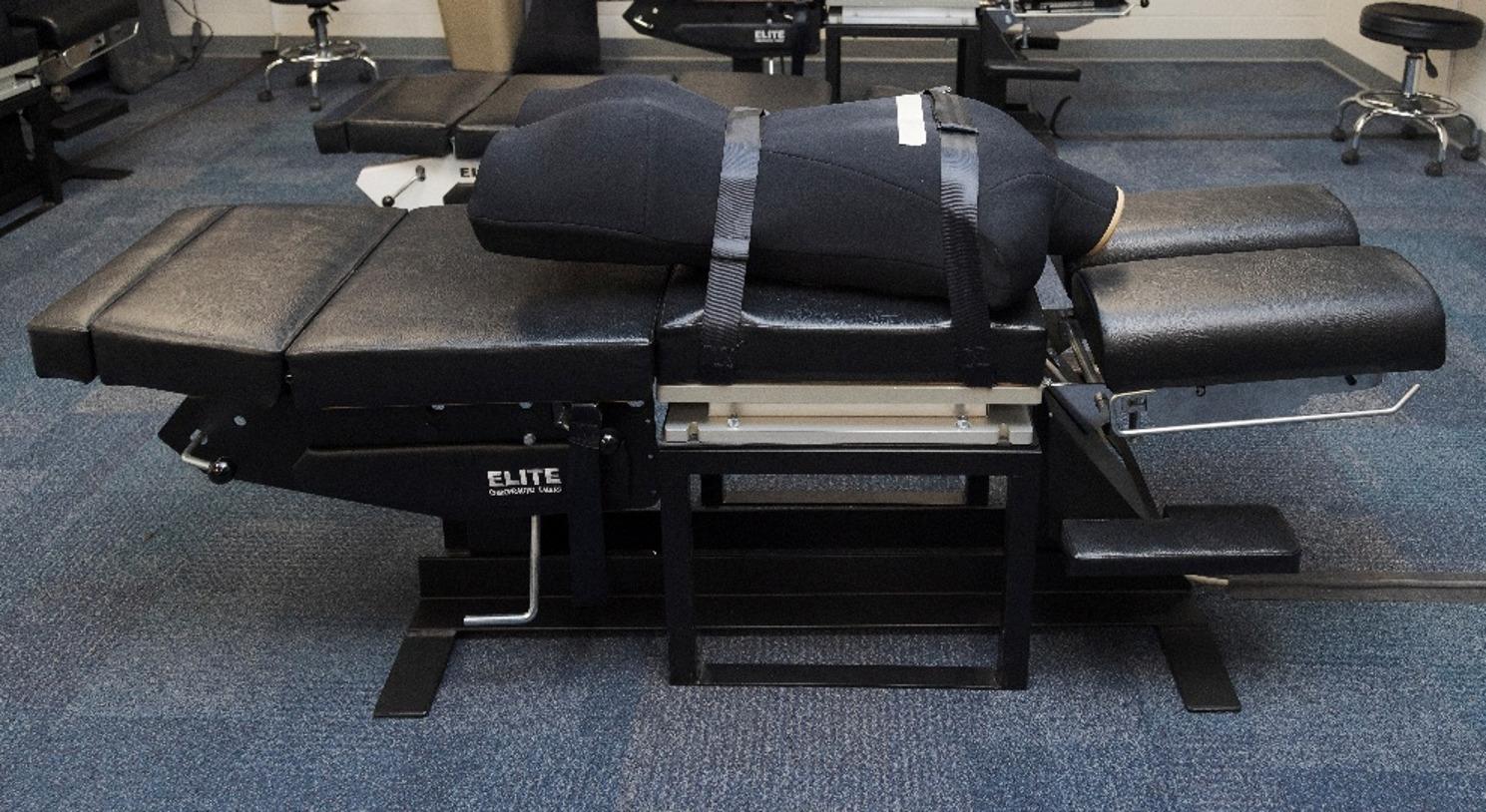
Experimental set up

The HAM^®^ was placed in a prone position on the FSTT^®^ and restrained by straps. The upper border of the shoulders was aligned with the edge of the table’s thoracic section, and a tape was placed at the mid-thoracic level (14in from the top of the headless HAM^®^) to standardize the location of SMT application (Fig. 1).

### Study overview

Data collection took place at CMCC’s FSTT^®^ Laboratory. All eligible participants who agreed to participate completed an online survey recording their individual data (e.g., demographic, anthropometric information - sex, age, BMI [calculated from height and weight], and year of study). Participants who participated as “providers” were then offered the opportunity to warm up by practicing any SMT force-time characteristics on the HAM^®^ and having the FSTT^®^ graphic feedback for 5 min. After the warm-up period, FSTT^®^ graphic feedback was blinded to both “providers” and “receivers” until the end of all trials.

**Fig. 2:**
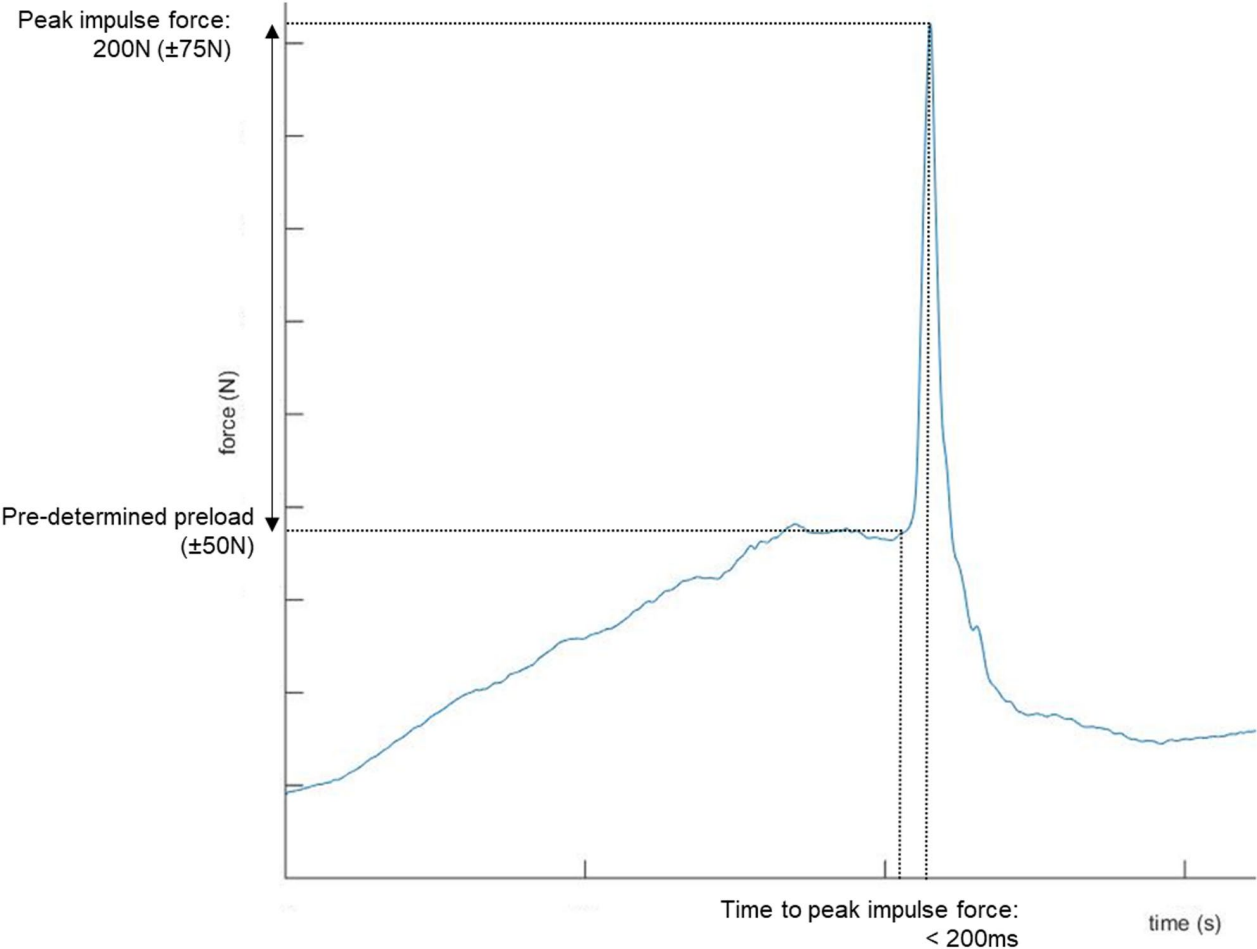
Spinal manipulative therapy force-time criteria

“Providers” then started the HAM^®^ phase of the study. First, they were asked to think of the HAM^®^ as a patient and to determine the preload force magnitude that they thought would be appropriate. After establishing the preload force magnitude, the “provider” performed 5 trials of posterior-to-anterior thoracic SMT to the HAM^®^, using their preferred hand contact, targeting the following parameters of force-time characteristics (Fig. 2):

- The pre-determined preload (± 50 N).
- A peak impulse force of 200 N (± 75 N).
- With a time to peak force of < 200ms.

Specifically, “providers” were instructed to apply “the same preload they established within ± 50 N, 200 N peak impulse force within ± 75 N in less than 200ms”. In other words, preload force was participant-defined (based on their judgment) and then used as the individualized target for subsequent trials. “Providers” who performed SMT on the HAM^®^ within all pre-specified parameters of the force-time characteristics in at least 4 out of the 5 trials (i.e., met the criteria for proficiency on the HAM^®^) continued to the next phase of the study (human phase).

Between the HAM^®^ and human phases, participants were asked to complete an online survey about their previous experience practicing manual therapy with the “receiver”. Then, participants were asked to define the preload force magnitude that they thought was appropriate for the human receiver (i.e., participants who agreed to participate as “human receivers”) by applying an initial gradual force until the point where they felt they had reached maximal displacement of the spinal segment using the least amount of force possible to reach that limit [13]. Once the participant established the preload force magnitude, they were asked to apply 3 SMT trials, targeting the same pre-specified parameters for the force-time characteristics as described above.

Immediately after applying the SMT to the “human receiver”, “providers” were asked to complete one last survey, which included anxiety levels (visual analogue scale; 0 = no anxiety, 100 = maximum anxiety) related to applying the pre-specified SMT force-time characteristics. It also included open-ended questions asking if participants believed they were able to replicate the SMT force-time characteristics to the human receiver (Yes/Partially/No; if Partially/No, explain why), if they believed the SMT (with the specific force-time characteristics) was appropriate for that “human receiver” (Yes/No; if No, why not), and their perceptions of differences between the HAM^®^ and the “human receiver”. All surveys and data collection forms can be found as a Supplementary file.

### Data processing

All SMT forces were measured at the participant-table interface (FSTT^®^ readings). The standard algorithms of the FSTT^®^ software were used to automatically identify and extract specific SMT force-time characteristics [14]. The following force-time characteristics were extracted for each SMT trial:

- Preload force [N]: the maximum force observed just before the impulse, which is an inflection in the force-time curve.
- Peak impulse force [N]: the maximum force reached after the preload phase before a subsequent decrease in force (i.e., total peak force minus preload force).
- Time to peak force [ms]: the time between the impulse initiation and peak impulse force.

A graphical representation of the force-time data and automatically extracted output for each SMT trial was reviewed by MF to verify that key force-time characteristics (such as preload force and peak impulse force) were correctly identified. In cases where the automated detection failed (e.g., either did not or incorrectly identified preload and/or peak impulse values), the relevant points on the force-time graph were manually identified by consensus between MF and GC and annotated within the FSTT^®^ software (*n* = 23 trials out of 633 trials).

### Outcomes

The primary outcome was the “provider” proficiency rate in the HAM^®^ and human phases. Participants could be proficient only with the HAM^®^ or with both the HAM^®^ and human. A “provider” was deemed to be proficient in the HAM^®^ phase if they successfully achieved the criteria for all the pre-specified SMT force-time characteristics (i.e., preload force (± 50 N), peak impulse force of 200 N (± 75 N), time to peak force < 200ms) in at least 4 out of the 5 trials performed on the HAM^®^. Those who were proficient in both the HAM^®^ and human phases satisfied the criteria for proficiency on the HAM^®^ and achieved the same criteria for all the pre-specified SMT force-time characteristics in at least 2 out 3 trials performed on the human phase. Dichotomous definitions (meeting vs. not meeting the proficiency criteria) were used as outcome variables in the analyses.

### Sample size calculation

Sample size was estimated using R version 4.3 and R-Studio version 2023.12 [15]. Expecting an a priori proficiency rate of 50% in the human phase of the study, with a lower confidence limit of 15%, an alpha value of 0.05, and a beta error of 0.8, a total sample of 42 participants was needed in the human phase (i.e., meeting the proficiency criteria in the HAM^®^ phase) (https://osf.io/waks4/). To progress to the human phase in this study, participants had to be proficient at applying SMT to the HAM^®^. Based on a previous study [16], approximately 30% of participants were able to apply SMTs to the HAM^®^ within the accepted ranges for the pre-specified force-time characteristics. Therefore, this study targeted to recruit a maximum of 142 participants as “providers”.

### Statistical analysis

#### Descriptive analysis

Descriptive statistics were used to characterize the participant sample. “Providers” were stratified between those who: (1) did not meet the proficiency criteria on the HAM^®^ phase, (2) met the criteria on the HAM^®^ phase but did not meet the criteria on the human phase, and (3) met the criteria on both the HAM^®^and human phases. Demographics data and SMT force-time characteristics were analyzed separately for participants stratified to each group (1–3 described above). The distributions of between target and achieved preload force, peak impulse force, and time to peak force were visualized using violin plots with embedded box plots and jitter points along with indications of meeting the criteria. Each SMT trial was depicted individually (i.e., 5 trials in HAM^®^ and 3 in human).

#### Status of meeting proficiency criteria and comparisons

The proportion of participants who met the proficiency criteria during the human phase was calculated, including 95% confidence intervals. Chi-square or Fisher’s Exact tests were used to investigate potential associations between participant characteristics and meeting the proficiency criteria on the human phase. A revised analysis plan was uploaded to OSF prior to any data analysis being conducted (https://osf.io/waks4/files/wp5jd). Specifically, per the analysis plan logistic regression modeling was originally planned, but due to the unbalanced and relatively small sample distribution in the human phase (i.e., *n* = 13 in the group that did not meet the proficiency criteria in the human phase), regression assumptions could not be met. Therefore, we opted to use Pearson’s Chi-squared test (for 5 or more counts) or Fisher’s exact test (for less than 5 counts) for categorical data, and Wilcoxon rank sum test for continuous data. Participant ratings of being able to replicate the SMT force-time characteristics to the human receiver and anxiety were also analyzed in this step.

A supplementary analysis was conducted that compared participants’ beliefs of being able to replicate the SMT force-time characteristics to the human receiver with their actual measured performance in the human phase following the identical analytical approach as described above.

#### Participants perceptions

An inductive content thematic analysis was used to explore participants’ perceptions regarding their ability to replicate the SMT force-time characteristics in the human phase, if they believed the SMT (with the specific force-time characteristics) was appropriate for the human receiver, and their perceived difference between the HAM^®^ and the human receiver. This approach drew on principles of both content analysis and thematic analysis and enabled both the assessment of themes and the interpretation of their significance within the context of participant perceptions. Two reviewers (KW and AG) independently coded each open-ended response to each question. Meetings were conducted after coding batches of 15–20 responses, where codes were reviewed, coding consensus was reached and then refined into thematic categories and definitions. A third reviewer (MF) participated in coding discussions for contextualization, reviewed coding decisions, and addressed discrepancies. Representative quotes with identifiers were used to illustrate the perceptions of different participants. Microsoft Excel spreadsheets (Microsoft Corporation, USA) were used for individual coding and combined coding decisions and generated the primary database and coding structure. Once data analysis was complete, all investigators reviewed the qualitative and quantitative components.

## Results

A total of 95 participants were included as “providers” (i.e., providing the SMT) and provided usable data (100% of those tested). Fifty students met the proficiency criteria on the HAM^®^ phase, and 37 of those students met the proficiency criteria on the human phase. This represents approximately 39% of the total recruited sample (37 out of 95) who achieved proficiency across both phases.

**Fig. 3:**
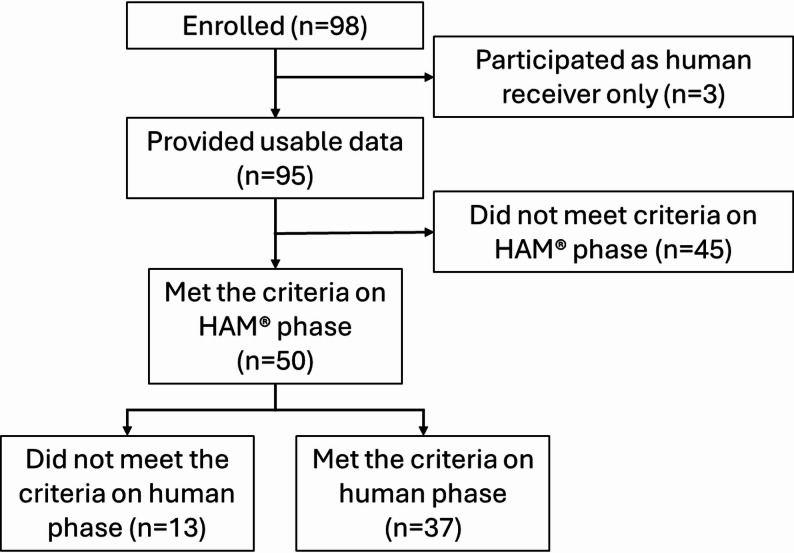
Participant flowchart

A participant flowchart is presented in Fig. 3.

Fifty-three participants were female (56%) with a median age of 25 years and a BMI ~ 25 kg/m^2^. Forty-four participants (46%) were in their second year of study. Participant characteristics did not differ widely between meeting the proficiency criteria or not (Table 1).

**Table 1:** Participant characteristics stratified by status of meeting or not the proficiency criteria in each study phase

Participant characteristic	Overall *N* = 95	Not meeting criteria on HAM^®^ phase *N* = 45	Meeting criteria on HAM^®^ phase, Not meeting criteria on human phase *N* = 13	Meeting criteria on human phase *N* = 37
Sex				
Female	53 (56%)	24 (53%)	8 (62%)	21 (57%)
Male	42 (44%)	21 (47%)	5 (38%)	16 (43%)
Age (years)	25 (24, 25)	25 (24, 26)	24 (23, 25)	25 (24, 25)
BMI (kg/m^2^)	24.8 (22.5, 26.8)	24.4 (22.3, 26.8)	24.4 (23.0, 25.2)	24.9 (23.5, 26.5)
Year of study				
1st year	4 (4.2%)	0 (0%)	2 (15%)	2 (5.4%)
2nd year	44 (46%)	23 (51%)	6 (46%)	15 (41%)
3rd year	33 (35%)	13 (29%)	5 (38%)	15 (41%)
4th year	14 (15%)	9 (20%)	0 (0%)	5 (14%)

*n* (%); Median (Q1, Q3)

*HAM*^®^ Human Analogue Mannequin, *SMT*  Spinal manipulation therapy, *BMI*  Body mass index

**Fig. 4:**
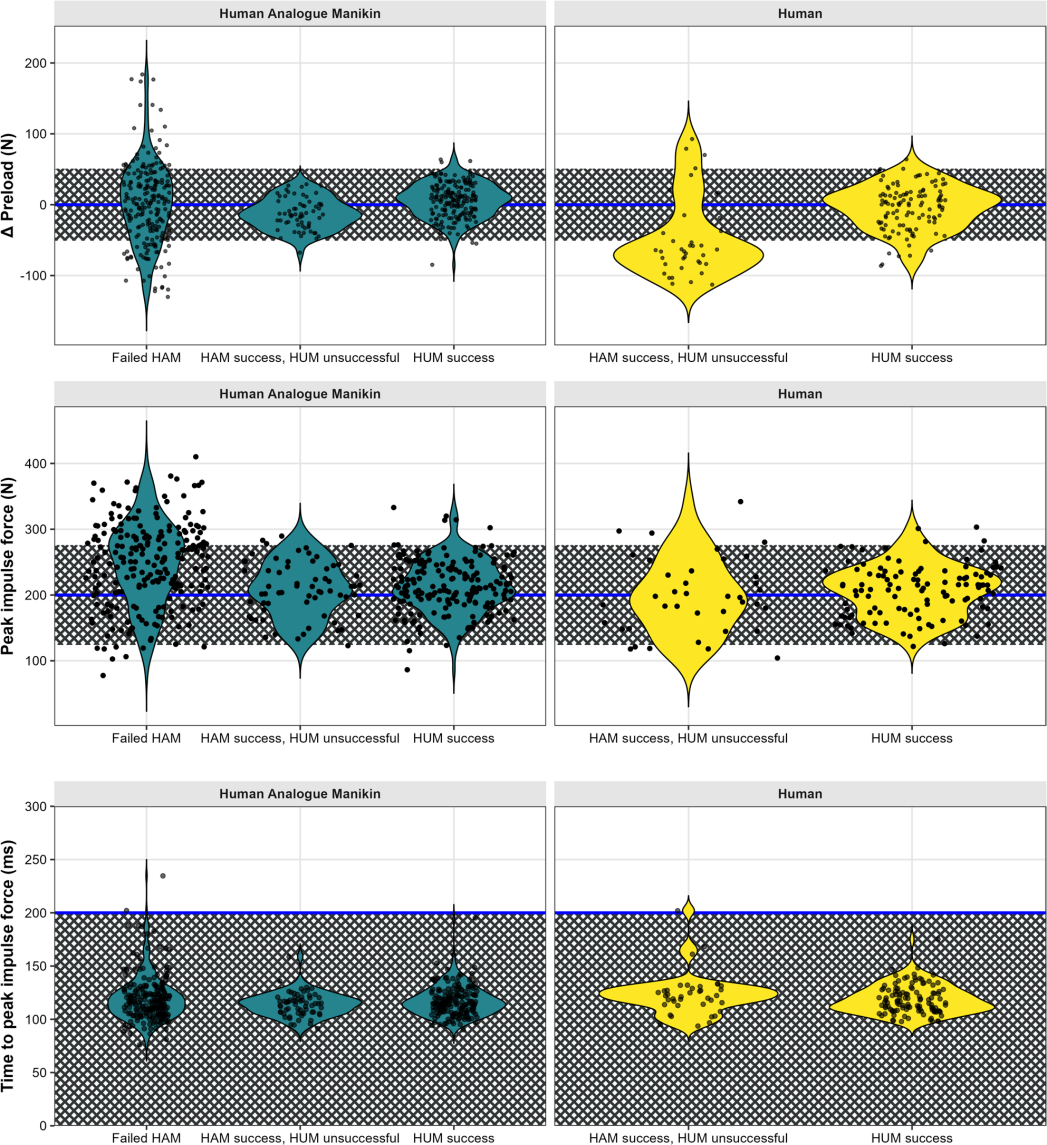
Force–time characteristics of SMT delivery, comparing HAM^®^ and human phases, stratified by meeting or not the proficiency criteria. HAM® = Human Analogue Mannequin, HUM = Human, SMT = Spinal manipulation therapy

Overall, across all participants, preload force showed greater variability, particularly in the human phase. Peak impulse force was more variable in HAM^®^ trials and time to peak was met across nearly all trials. Specifically, participants who did not meet the proficiency criteria on either the HAM^®^ or human phases exhibited more negative difference (Δ) preload values, indicating insufficient preload force relative to the target, particularly in human phase trials (Fig. 4). Peak impulse forces were more variable in HAM^®^ trials, with a broader spread across all statuses of meeting the proficiency criteria. Only a few trials not meeting the criteria on the human phase fell outside the target range for the peak impulse force. Nearly all trials achieved time to peak force under 200ms, regardless of proficiency group status (Fig. 4, Supplementary file).

### Status of meeting proficiency criteria and comparisons

Among the 50 participants who met the criteria on the HAM^®^ phase, 37 also met the criteria on the human phase, yielding a success rate of 74% with a 95% confidence interval ranging from 59% to 85%. There were no statistically significant differences in participant characteristics between those who met and those who did not meet the proficiency criteria on the human phase. Third- and fourth-year students, and participants with lower anxiety levels, were more commonly able to meet the criteria, but none of these differences reached statistical significance (all p-values > 0.2). Familiarity with the receiver and perception of SMT appropriateness was also higher among those who did not meet the proficiency criteria, though again not statistically significant (Table 2). Participants’ perception of whether they were able to replicate the pre-specified SMT force–time characteristics was not associated with their actual performance in the human phase (Table 3).

**Table 2:** Participant characteristic comparing ability to meet the proficiency criteria on the human phase

Participant characteristic	Not meeting criteria on human phase *N* = 13	Meeting criteria on human phase *N* = 37	*P*-value
Sex			0.8
Female	8 (62%)	21 (57%)	
Male	5 (38%)	16 (43%)	
Year of study			0.4
1st year	2 (15%)	2 (5.4%)	
2nd year	6 (46%)	15 (41%)	
3rd year	5 (38%)	15 (41%)	
4th year	0 (0%)	5 (14%)	
BMI (kg/m^2^)	24.4 (23.0, 25.2)	24.9 (23.5, 26.5)	0.6
Familiar with the human [Yes]	9 (69%)	19 (51%)	0.3
Anxiety [0-100]	33 (10, 60)	25 (4, 50)	0.6
Considered SMT appropriate	7 (54%)	10 (27%)	0.1

*n* (%); Median (Q1, Q3)

*SMT*  Spinal manipulation therapy, *BMI*  Body mass index

**Table 3:** Participants’ perceptions that they met the proficiency criteria on the human phase

Considered SMT replicated	Not meeting criteria on human phase *N* = 13	Meeting criteria on human phase *N* = 37	*P*-value
Partially or not replicated	8 (62%)	22 (59%)	0.9
Replicated	5 (38%)	15 (41%)	

n (%)

*SMT*  Spinal manipulation therapy

### Participants’ perceptions

#### Ability to replicate the SMT force-time characteristics to the human receiver

Responses from 31 participants who indicated they believed they were partially or not able to replicate the SMT force-time characteristics on the humans described a multifaceted experience indicating that their performance was influenced by internal processes within themselves, as well as perceived differences between the HAM^®^ and humans.

Participants’ reflections focused on how internal factors, such as tactile feel (*“The end feel on a mannequin feels different compared to a human so trying to replicate the manipulation felt a bit different” [P33]*) and previous experience (*“I haven’t adjusted this patient before so I was more focused on having a comfortable adjustment and establishing preload specific for the patient. […]” [P05]*), influenced their SMT performance. Participants also reflected on their own mental and emotional factors, acknowledging that the transition from practicing on the HAM^®^ to performing on a human introduced a layer of psychological complexity: *“[…] I also feel there is cognitive dissonance when trying to complete a 200 N force on a human*,* because experience has told us that people likely need more to achieve cavitation. There is also the component of anxiety*,* that you “can’t take it back and try again” that occurs when you work with humans that you don’t experience with a manikin” [P84].*

Participants also emphasized that while some SMT factors are straightforward on the HAM^®^, they become more complex in the human receiver: *“I think the preload felt different on this particular human. Also their layers of clothing changed the skin slack element” [P10]*.

Most participants attributed the challenge of replicating the pre-specified SMT characteristics on human receivers to the differences between the HAM^®^ and humans. A recurring theme was the Human body factors, including the variability related to tissue texture and breath (*“ […] there are different texture of backs such as tender*,* stiff*,* tight that cannot be replicated. Breathing in and out also contributes to an individual preload” [P77]*) as well as the individuality of each person: *“each individual patient/person requires specific force-time characteristics*,* different from practising on a mannequin” [P93].* Within Mannequin factors, participants highlighted how the HAM^®^ stiffness and structure could influence their performance: *“I find the manikins much less pliable than the human spine and stiffer. The manikins do not have all the body parts to the human body*,* and so they move differently (tilt) when you add preload” [P03].*

### Perceptions on the SMT pre-specified characteristics appropriateness

From 35 responses, participants’ reflections centered primarily around SMT Characteristics, with supporting themes of Human Factors, Provider Factors, and Goals/Effects expanding on their rationale.

The dominant theme, SMT Characteristics, captured participants’ belief that the pre-specified force was insufficient for the individual they were working with. Many felt that the thrust lacked the necessary peak impulse force: *“I didn’t think 200 N was enough for this person” [P14]*.

Human Factors were mentioned to explain why the pre-specified SMT characteristics may not have been appropriate: *“His body is bigger than average male therefore I needed more force to actually adjust. I did feel like 200 N was not sufficient to make a difference” [P52]*.

Provider Factors also seemed to influence participants’ beliefs, particularly their tactile feel and previous experience, which influenced participants’ perceptions on SMT force appropriateness: *“I think they need more force (based on working with them before).” [P27]*.

Finally, the theme of Goals/Effects reflected participants’ expectations regarding SMT outcomes, such as audible pop or perceived therapeutic effects: *“Felt like they needed more force to achieve therapeutic effect and to get cavitations.” [P32]*.

### Perceived difference between the mannequin and the human receiver

Based on 50 responses, participants again emphasized the dynamic and variable nature of the Human body. Overall, humans were perceived to be more pliable than the mannequins: *“The persons spine is more pliable and the body as a whole moves differently compared to the spine […]” [P44]*, however some perceived humans to be stiffer *“I had to focus more on following the breath and the patient was more stiff than the manikin” [P24].* The presence of breathing was also noted *“The way their back feels compared to the manikin I different. Humans are more dynamic and there is the skin and breathing component” [P61].*

Among the Mannequin factors, it was generally perceived to have greater stiffness (*“[…] The mannequin felt more stiff” [P15]*) and rigidity (*“The manikin was a lot more rigid […]” [P31]*) than human receivers. The way the HAM^®^ moved was also noted: *“The person was more squishy and rocked back and forth less” [P20]*.

Participants also noted some SMT factors, with preload and skin slack being frequently perceived differently on a human: *“Different tissue feeling- more skin slack. Perceived preload was different as I found preload with breath not just pressure.” [P30]*; *“More preload needed due to the manikin not having as much give whereas the person you sink deeper to obtain the preload” [P34].*

Finally, participants noted again internal factors and described feeling less confident *(“I used less preload potentially*,* since it is an actual person I don’t feel as confident to put my body weight into the preload as much” [P43])* and more anxious *(“there was more anxiety over adjusting a person versus the manikin” [P12]*) when applying SMT to a human.

## Discussion

This study found that 74% of students who proficiently applied specific SMT force-time characteristics to the HAM^®^ were also proficient when performing SMT on human receivers. Participants who were unable to replicate SMT force-time characteristics both in the HAM^®^ and particularly in humans most often struggled with achieving a pre-specified target preload force. Proficiency of performing SMT on a human receiver was not statistically associated with any student characteristics. This study marks an important step towards a better understanding of how motor skills learned in controlled environments may (or may not) translate to the real-world, contributing to advancing our knowledge on simulation-based education in manual therapy.

The finding that most students were able to replicate specific SMT force-time characteristics practiced on the HAM^®^ to human receivers suggests that people who demonstrate SMT proficiency, as defined in the current study, on a mannequin are likely also proficient when applying SMT on a human. This contrasts with Duquette et al. [13], who found that while a 1-hour training session with objective force feedback significantly decreased the error from a target cervical SMT peak force on mannequins, this improvement was not observed in SMT applied to humans [13]. One possible explanation is that Duquette et al. [13] measured performance immediately after feedback-based training, whereas our study did not provide any feedback or additional practice. It is possible that students who achieve proficiency without feedback may have developed more robust motor strategies that facilitate transferring SMT motor skills from mannequins to humans. This is consistent with previous studies observing that training involving simulators can enhance both knowledge acquisition and skill development in medical students [8, 17, 18]. Specifically, simulation has been observed to support and facilitate the learning of technical and procedural skills (such as surgical procedures and clinical interventions), which are essential for clinical practice of all healthcare professionals. Previous studies involving medical students showed that repeated practice with simulators led to improved performance and greater skill retention compared to traditional teaching methods [8, 17, 19]. Additionally, using simulators such as mannequins (as opposed to fellow students or patients), has been described as a way to provide a controlled and low-risk learning environment for students to develop their skills and confidence, contributing to reducing the incidence of errors to actual patients and enhancing patient safety [8, 17, 19].

Participants who did not meet the proficiency criteria in replicating SMT force-time characteristics commonly struggled with achieving the preload force within the pre-specified limits, often applying a preload force below the target. While this pattern was less pronounced in the HAM^®^ phase, it became noticeable during the human phase. This could be related to the reliance on tactile perception for determining preload [13, 20]. This subjective process introduces variability, particularly when transitioning from a more rigid and standardized mannequin to a more deformable human body with differences in tissue compliance and breathing, as emphasized by our qualitative findings. This variability in preload force is aligned with findings from previous studies [16, 21]. Specifically, Nim et al. [15] observed that although students could generally achieve a target preload force, variability persisted across trials [16]. Conversely, the review conducted by Gorrell et al. [6] reported preload forces applied on mannequins during SMT to the thoracic spine did not vary much, ranging between 137 and 172 N [6]. However, most studies included by Gorrell et al. [6] were descriptive in nature and did not investigate achieving a target preload force over multiple SMT applications. In combination with the results from Nim et al. [15, 16], findings from this study may suggest that being able to repeatedly achieve a target preload force is an area for attention during manual therapy training, particularly given its reliance on perceptual judgment and the influence of contextual factors such as human receiver characteristics.

There was no participant characteristic that was significantly associated with successful replication of SMT force-time parameters between the HAM^®^ and human receivers. The small number of participants not meeting the proficiency criteria on the human phase requires caution in generalizing these findings. Despite that, Nim et al. [15] showed that students’ confidence is positively associated with their ability to modulate SMT force-time characteristics, particularly in being able to achieve a pre-specified preload and peak impulse forces on mannequins [16]. This relationship was not influenced by demographic or anthropometric characteristics [22] and suggests that motor skill performance is influenced by mental and emotional factors rather than providers’ demographic and anthropometric characteristics. This is supported by the current study’s qualitative results where participants described how psychological factors, including anxiety, affected their perceived ability to replicate the pre-specified SMT on human receivers. These reflections align with the collaborative nature of motor and cognitive learning observed in medical training, where emotional and mental states are known to influence performance [23]. These findings indicate that manual therapy training may benefit from focusing not only on technical skills but also on the emotional and cognitive dimensions of skill acquisition.

Interestingly, participants’ perceptions of whether they successfully replicated the SMT force-time characteristics did not align with their actual SMT performance on the human phase. This disconnect may reflect a mental conflict between meeting the pre-specified SMT force-time targets and participants’ own beliefs about what SMT force-time characteristics were appropriate for a given individual. Indeed, qualitative responses indicated that the pre-specified 200 N peak impulse force was perceived as insufficient to achieve desired outcomes (e.g., audible “pop” or therapeutic benefit). Therefore, it is possible that participants may have prioritized these clinical expectations over the experimental criteria, highlighting the tension between technical proficiency and contextual judgment. Another contributing factor may be multiple parameters (preload, peak impulse force, and time to peak) that needed to be met for the proficiency criteria. This multidimensional requirement could have made participants uncertain about whether they truly achieved proficiency.

The qualitative component of this study further highlighted participants’ perceptions of the nuanced challenges involved in transferring SMT force-time characteristics from mannequins to humans. Across all three open-ended questions, participants described a complex relationship of factors influencing their performance, including differences in structural properties, technical aspects of SMT application, and internal provider-related factors such as anxiety and tactile feel. These findings are aligned with the dynamical systems theory of motor learning, which views motor performance as an emergent phenomenon shaped by the interaction of individual (e.g., anxiety, experience), task (e.g., pre-specified SMT force-time targets), and environmental constraints (e.g., human vs. mannequin structural properties) [24–26].

From this perspective, motor skill proficiency is not the result of replicating fixed or pre-programmed patterns, but rather the outcome of adaptive responses to varying conditions [24–26]. For example, participants noted that human variability, such as differences in stiffness and breathing, introduced environmental constraints that required adjustments in preload and impulse force and thrust, and challenged participants to adjust their motor output in real time [24–26]. Similarly, internal factors like emotional state and tactile perception represent individual constraints that influence performance, demonstrating how performance is context-sensitive [24–26]. This is consistent with *ecological dynamics approach*, which emphasize perception-action coupling and the importance of practicing in environments that reflect the complexity of real-world clinical scenarios [25–28].

These findings align with Gamborg et al. (2024), who emphasized the interdependence of technical and non-technical skills in health education [28], and with Wulf and Lewthwaite’s (2016) OPTIMAL theory, which highlights the role of attentional focus and motivational states in optimizing motor performance [29]. Together, these perspectives support that manual therapy training should incorporate constraint-rich learning environments that promote adaptability, variability, and self-organization. Specifically, rather than focusing solely on achieving predefined force-time targets, training could consider encouraging learners to explore a range of movement strategies and develop flexible motor solutions that can be transferred across contexts. This approach may better support the development of robust, context-sensitive manual therapy skills.

### Strengths and limitations

One of the key strengths of this study is its mixed-methods design, which allowed for a comprehensive exploration of students learning to perform SMT force-time characteristics using both the quantitative performance outcomes and the qualitative perceptions. Consequently, this study provides a nuanced understanding of the factors perceived by students to influence their motor skill transfer from simulation to human application. This approach enabled the identification of psychological and perceptual factors that would not be captured through quantitative measures alone.

Some limitations should be acknowledged. First, the study was conducted within a single educational institution, which may limit the generalizability of the findings to other training environments or student populations. Second, while the pre-specified SMT force-time parameters used to define proficiency in this study were selected based on existing literature and expert consensus, there is currently no universally accepted standard for what constitutes proficiency. Consequently, our results should be interpreted with caution and within the context of the predefined proficiency criteria, as changes in the proficiency criteria will likely influence the results. Third, qualitative responses indicated that the chosen peak impulse force (200 N) may have been perceived as insufficient for some human receivers, suggesting that the force-time targets may not have fully reflected clinical realities. Fourth, only participants who met the proficiency criteria on the HAM^®^ were allowed to progress to the human phase. Although 74% of participants who achieved proficiency on the HAM^®^ were also proficient on humans, this represented only 39% of the total recruited sample. This percentage (39%) reflects the proportion of participants who achieved proficiency across both phases, not the success rate among those who attempted the human phase and should be interpreted with caution. This approach allowed us to focus on the transfer of proficiency from simulation to real-world application. However, it also precluded evaluation of SMT performance on humans among those who did not meet proficiency criteria on the HAM^®^, limiting our conclusions about their potential proficiency on humans. Fifth, this study focused on SMT force-time characteristics as a proxy for technical proficiency and findings should not be extrapolated to global clinical competence or therapeutic effectiveness. Sixth, anxiety was assessed using a self-reported visual analogue scale, which is not a validated measurement tool for this construct. While anxiety scores did not show a significant association with proficiency, qualitative responses suggest it may influence motor performance. Additionally, participants’ perceptions were captured via open-ended questions on an electronic survey. While this allowed for an initial exploration, future studies should consider conducting qualitative interviews for more in-depth data. Responses to the open-ended questions were recorded and consisted of short phrases of few words to full paragraphs in length. Therefore, data adequacy cannot be ascertained. Finally, given the observed discrepancy between qualitative and quantitative influence of anxiety, future research could benefit from incorporating validated psychometric instruments to quantitatively assess constructs such as anxiety and their relationship to motor performance.

## Conclusion

Findings from this study show that most students (74%) who were proficient in applying pre-specified SMT force-time characteristics on a mannequin could replicate those characteristics on humans. However, non-proficient students commonly struggled with preload force, and no participant characteristics were statistically associated with successful transfer. Qualitative findings reflect the complexity of manual therapy motor skill development, highlighting the influence of anatomical variability, technical challenges, and psychological factors such as anxiety. These findings highlight the potential value of using human simulators in manual therapy education, while also suggesting the need for future research to explore andragogical strategies that incorporate the cognitive and emotional dimensions of motor learning.

## Supplementary Information

Supplementary Material 1.

Supplementary Material 2.

Supplementary Material 3.

Supplementary Material 4.

## Data Availability

The datasets used and/or analyzed during the current study are available from the corresponding author on reasonable request.

## Abbreviations

SMT: Spinal manipulative therapy
FSTT: Force Sensing Table Technology
HAM: Human analogue mannequin
GRAMMS: Good Reporting of A Mixed Methods Study
CMCC: Canadian Memorial Chiropractic College
REB: Research ethics board
N: Newtons
OSF: Open Science Framework
ms: Milliseconds
N/ms: Newtons per millisecond
BMI: Body mass index
