# Supplementary material for: From mannequins to humans – are manual therapy motor skills transferable? A mixed-methods study
Source: BMC Med Educ. 2026 Feb 14;26:459. doi: 10.1186/s12909-026-08806-7 (PMC13011724; doi:10.1186/s12909-026-08806-7)
Supplement: Supplementary file 1 — Supplementary Material 1. [file 12909_2026_8806_MOESM1_ESM.docx]

Supplementary File

Table 1: **Student SMT Pass HAM Matrix**

● = Pass, ○ = Fail

|  |  | Test 1 | | | Test 2 | | | Test 3 | | | Test 4 | | | Test 5 | | |
| --- | --- | --- | --- | --- | --- | --- | --- | --- | --- | --- | --- | --- | --- | --- | --- | --- |
|  | Passed to HUM | Preload | Impulse | Time | Preload | Impulse | Time | Preload | Impulse | Time | Preload | Impulse | Time | Preload | Impulse | Time |
| 1 | Yes | ● | ● | ● | ● | ● | ● | ● | ● | ● | ● | ● | ● | ● | ● | ● |
| 2 | Yes | ● | ● | ● | ● | ● | ● | ● | ● | ● | ● | ● | ● | ● | ● | ● |
| 3 | No | ● | ● | ● | ● | ● | ● | ● | ○ | ● | ● | ○ | ● | ● | ● | ● |
| 4 | No | ● | ● | ● | ● | ○ | ● | ● | ○ | ● | ● | ● | ● | ● | ○ | ● |
| 5 | No | ○ | ● | ● | ● | ○ | ● | ○ | ● | ● | ● | ● | ● | NA | NA | NA |
| 6 | Yes | ● | ● | ● | ● | ● | ● | ○ | ● | ● | ● | ● | ● | ● | ● | ● |
| 7 | Yes | ● | ● | ● | ● | ● | ● | ● | ● | ● | ● | ● | ● | ● | ● | ● |
| 8 | Yes | ● | ● | ● | ● | ● | ● | ● | ● | ● | ● | ● | ● | ● | ● | ● |
| 9 | Yes | ● | ● | ● | ● | ● | ● | ● | ● | ● | ● | ● | ● | ● | ● | ● |
| 10 | No | ● | ● | ● | ● | ● | ● | ● | ● | ● | ● | ● | ● | ● | ○ | ● |
| 11 | Yes | ● | ● | ● | ● | ● | ● | ● | ● | ● | ● | ● | ● | ● | ● | ● |
| 12 | No | ● | ○ | ● | ● | ○ | ● | ● | ● | ● | ○ | ● | ● | ● | ● | ● |
| 13 | No | ○ | ● | ● | ● | ● | ● | ● | ○ | ● | ● | ○ | ● | ● | ● | ● |
| 14 | No | ● | ○ | ● | ● | ○ | ● | ● | ○ | ● | ● | ○ | ● | ● | ○ | ● |
| 15 | No | ○ | ○ | ● | ○ | ○ | ● | ○ | ● | ● | ○ | ○ | ● | ○ | ● | ● |
| 16 | No | ● | ● | ● | ● | ○ | ● | ● | ● | ● | ● | ○ | ● | ● | ○ | ● |
| 17 | Yes | ● | ○ | ● | ● | ● | ● | ● | ● | ● | ● | ● | ● | ● | ● | ● |
| 18 | Yes | ● | ● | ● | ● | ● | ● | ● | ● | ● | ● | ● | ● | ● | ● | ● |
| 19 | No | ● | ● | ● | ● | ● | ● | ○ | ● | ● | ○ | ○ | ● | ○ | ● | ● |
| 20 | Yes | ● | ○ | ● | ● | ● | ● | ● | ● | ● | ● | ● | ● | ● | ● | ● |
| 21 | Yes | ● | ● | ● | ● | ● | ● | ● | ● | ● | ● | ● | ● | ● | ● | ● |
| 22 | Yes | ● | ● | ● | ● | ● | ● | ● | ● | ● | ● | ● | ● | ● | ○ | ● |
| 23 | No | ● | ● | ● | ● | ● | ● | ● | ● | ● | ○ | ● | ● | ○ | ● | ● |
| 24 | Yes | ● | ● | ● | ● | ● | ● | ● | ● | ● | ● | ● | ● | ● | ● | ● |
| 25 | No | ● | ● | ● | ● | ● | ● | ● | ● | ● | ● | ○ | ● | ● | ● | ● |
| 26 | No | ○ | ● | ● | ○ | ● | ● | ○ | ● | ● | ○ | ● | ● | ○ | ● | ● |
| 27 | No | ● | ○ | ● | ● | ○ | ● | ● | ○ | ● | ○ | ○ | ● | ● | ○ | ● |
| 28 | No | ● | ● | ● | ● | ● | ● | ● | ● | ● | ○ | ● | ● | ○ | ● | ● |
| 29 | No | ● | ● | ● | ● | ● | ● | ● | ○ | ● | ● | ● | ● | ● | ○ | ● |
| 30 | Yes | ● | ● | ● | ● | ● | ● | ● | ● | ● | ● | ● | ● | NA | NA | NA |
| 31 | Yes | ● | ● | ● | ● | ● | ● | ● | ● | ● | ● | ● | ● | ● | ● | ● |
| 32 | Yes | ● | ● | ● | ● | ● | ● | ● | ● | ● | ● | ● | ● | ○ | ● | ● |
| 33 | Yes | ● | ● | ● | ● | ● | ● | ● | ● | ● | ○ | ● | ● | ● | ● | ● |
| 34 | No | ● | ● | ● | ○ | ● | ● | ○ | ● | ● | ● | ● | ● | ● | ● | ● |
| 35 | Yes | ○ | ● | ● | ● | ● | ● | ● | ● | ● | ● | ● | ● | ● | ● | ● |
| 36 | No | ● | ○ | ● | ○ | ○ | ● | ● | ● | ● | ○ | ● | ● | ● | ● | ● |
| 37 | Yes | ● | ○ | ● | ● | ● | ● | ● | ● | ● | ● | ● | ● | ● | ● | ● |
| 38 | No | ● | ● | ● | ○ | ● | ● | ● | ● | ● | ○ | ○ | ● | ● | ● | ● |
| 39 | Yes | ● | ● | ● | ● | ○ | ● | ● | ● | ● | ● | ● | ● | ● | ● | ● |
| 40 | Yes | ● | ● | ● | ● | ● | ● | ● | ● | ● | ● | ● | ● | ● | ● | ● |
| 41 | No | ○ | ○ | ● | ● | ○ | ● | ● | ○ | ● | ● | ○ | ● | ○ | ● | ● |
| 42 | No | ● | ● | ● | ● | ○ | ● | ● | ● | ● | ● | ● | ● | ● | ○ | ● |
| 43 | Yes | ● | ● | ● | ● | ● | ● | ● | ● | ● | ● | ○ | ● | ● | ● | ● |
| 44 | Yes | ● | ● | ● | ● | ● | ● | ● | ● | ● | ● | ● | ● | ● | ○ | ● |
| 45 | No | ● | ● | ● | ● | ● | ● | ● | ● | ● | ● | ○ | ● | ● | ○ | ● |
| 46 | Yes | ● | ● | ● | ● | ● | ● | ● | ● | ● | ● | ● | ● | ● | ● | ● |
| 47 | No | ○ | ● | ● | ○ | ● | ● | ○ | ● | ● | ○ | ● | ● | ○ | ● | ● |
| 48 | No | ● | ● | ● | ○ | ○ | ● | ● | ● | ● | ● | ○ | ● | ● | ○ | ● |
| 49 | Yes | ● | ● | ● | ● | ○ | ● | ● | ● | ● | ● | ● | ● | ● | ● | ● |
| 50 | Yes | ● | ● | ● | ● | ● | ● | ● | ● | ● | ● | ● | ● | ● | ● | ● |
| 51 | Yes | ● | ● | ● | ● | ● | ● | ● | ● | ● | ● | ● | ● | ● | ● | ● |
| 52 | No | ○ | ● | ● | ○ | ● | ● | ○ | ○ | ● | ○ | ● | ● | ○ | ● | ● |
| 53 | No | ● | ● | ● | ● | ○ | ● | ● | ○ | ● | ● | ● | ● | ● | ○ | ● |
| 54 | No | ○ | ● | ● | ○ | ○ | ● | ○ | ○ | ● | ○ | ○ | ● | ○ | ● | ● |
| 55 | No | ● | ● | ● | ● | ● | ● | ● | ○ | ● | ○ | ○ | ● | ● | ● | ● |
| 56 | No | ○ | ● | ● | ○ | ● | ● | ○ | ○ | ● | ○ | ○ | ● | ● | ○ | ● |
| 57 | No | ● | ○ | ● | ● | ○ | ● | ○ | ○ | ● | ● | ○ | ● | ○ | ○ | ● |
| 58 | Yes | ● | ● | ● | ● | ● | ● | ● | ● | ● | ● | ● | ● | ● | ● | ● |
| 59 | Yes | ● | ● | ● | ● | ● | ● | ● | ● | ● | ● | ● | ● | ● | ● | ● |
| 60 | Yes | ● | ● | ● | ● | ● | ● | ● | ● | ● | ● | ● | ● | ● | ● | ● |
| 61 | Yes | ● | ● | ● | ● | ● | ● | ● | ● | ● | ● | ● | ● | ● | ● | ● |
| 62 | No | ● | ● | ● | ○ | ● | ● | ○ | ● | ● | ○ | ● | ● | ● | ● | ● |
| 63 | Yes | ● | ● | ● | ● | ● | ● | ● | ● | ● | ● | ● | ● | ● | ● | ● |
| 64 | No | ● | ● | ● | ● | ● | ● | ○ | ● | ● | ○ | ● | ● | ● | ● | ● |
| 65 | Yes | ● | ● | ● | ● | ● | ● | ● | ● | ● | ● | ● | ● | ● | ● | ● |
| 66 | No | ○ | ● | ● | ○ | ○ | ● | ○ | ● | ● | ○ | ○ | ● | ○ | ● | ● |
| 67 | No | ● | ● | ● | ● | ○ | ● | ● | ● | ● | ● | ○ | ● | ● | ● | ● |
| 68 | No | ○ | ● | ● | ○ | ● | ● | ● | ● | ● | ○ | ● | ● | ○ | ● | ● |
| 69 | Yes | ○ | ● | ● | ● | ● | ● | ● | ● | ● | ● | ● | ● | ● | ● | ● |
| 70 | Yes | ● | ● | ● | ● | ● | ● | ● | ○ | ● | ● | ● | ● | ● | ● | ● |
| 71 | No | ○ | ● | ● | ● | ● | ● | ○ | ● | ● | ○ | ● | ● | ● | ● | ● |
| 72 | Yes | ● | ● | ● | ● | ● | ● | ● | ● | ● | ● | ● | ● | ● | ● | ● |
| 73 | Yes | ● | ● | ● | ● | ● | ● | ● | ● | ● | ● | ● | ● | ● | ● | ● |
| 74 | No | ● | ○ | ● | ● | ● | ● | ● | ● | ● | ● | ● | ● | ● | ○ | ● |
| 75 | Yes | ● | ● | ● | ● | ● | ● | ● | ● | ● | ● | ● | ● | ○ | ● | ● |
| 76 | No | ● | ● | ● | ● | ● | ● | ● | ○ | ● | ● | ● | ● | ● | ○ | ● |
| 77 | Yes | ● | ○ | ● | ● | ● | ● | ● | ● | ● | ● | ● | ● | ● | ● | ● |
| 78 | No | ● | ● | ● | ● | ● | ● | ○ | ● | ● | ● | ● | ● | ○ | ● | ● |
| 79 | No | ● | ● | ● | ○ | ● | ● | ○ | ● | ● | ○ | ● | ● | ○ | ● | ● |
| 80 | Yes | ● | ● | ● | ● | ● | ● | ● | ○ | ● | ● | ● | ● | ● | ● | ● |
| 81 | Yes | ● | ● | ● | ● | ● | ● | ● | ● | ● | ● | ● | ● | ● | ● | ● |
| 82 | Yes | ● | ● | ● | ● | ● | ● | ● | ● | ● | ● | ● | ● | ● | ● | ● |
| 83 | Yes | ● | ○ | ● | ● | ● | ● | ● | ● | ● | ● | ● | ● | ● | ● | ● |
| 84 | No | ● | ○ | ● | ● | ○ | ● | ● | ○ | ● | ● | ○ | ● | ○ | ○ | ● |
| 85 | No | ○ | ● | ● | ○ | ○ | ● | ● | ● | ● | ● | ○ | ● | ● | ● | ● |
| 86 | Yes | ● | ● | ● | ● | ● | ● | ● | ● | ● | ● | ● | ● | ● | ● | ● |
| 87 | Yes | ● | ● | ● | ● | ● | ● | ● | ○ | ● | ● | ● | ● | ● | ● | ● |
| 88 | Yes | ● | ● | ● | ● | ● | ● | ● | ● | ● | ● | ● | ● | ● | ● | ● |
| 89 | Yes | ● | ● | ● | ● | ● | ● | ● | ● | ● | ● | ● | ● | ● | ● | ● |
| 90 | Yes | ● | ● | ● | ● | ● | ● | ● | ● | ● | ● | ● | ● | ● | ● | ● |
| 91 | Yes | ● | ● | ● | ● | ○ | ● | ● | ● | ● | ● | ● | ● | ● | ● | ● |
| 92 | No | ● | ○ | ● | ● | ○ | ● | ● | ○ | ● | ● | ○ | ● | ● | ○ | ● |
| 93 | No | ● | ○ | ● | ● | ● | ● | ● | ○ | ● | ● | ○ | ● | ● | ○ | ● |
| 94 | Yes | ● | ● | ● | ● | ● | ● | ● | ● | ● | ● | ● | ● | ○ | ● | ● |
| 95 | No | ● | ○ | ○ | ● | ● | ● | ● | ● | ● | ● | ● | ● | ● | ● | ○ |
