# Supplementary figures and images for: From mannequins to humans – are manual therapy motor skills transferable? A mixed-methods study
Source: BMC Med Educ. 2026 Feb 14;26:459. doi: 10.1186/s12909-026-08806-7 (PMC13011724; doi:10.1186/s12909-026-08806-7)

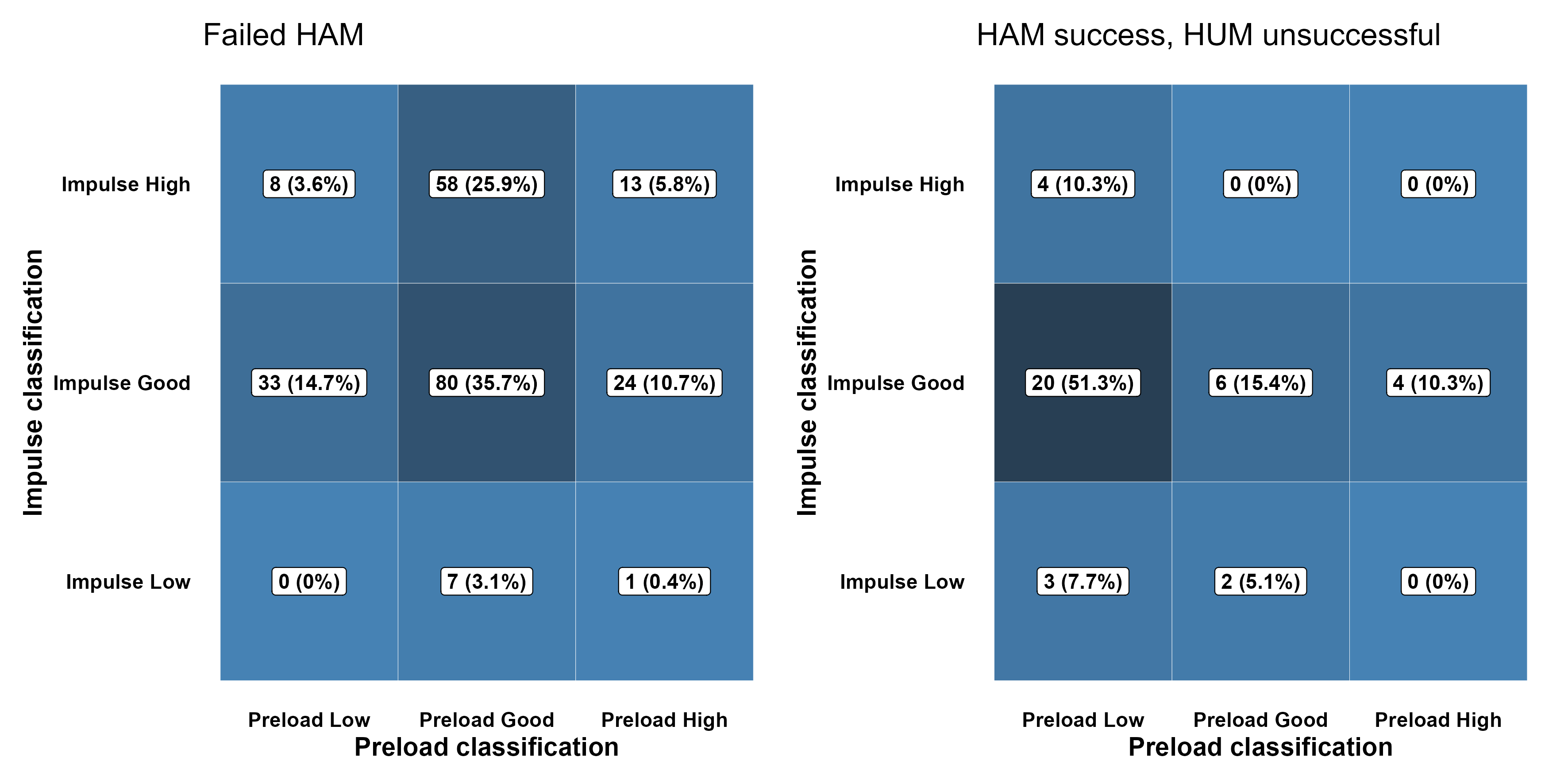

Supplement: Supplementary file 2 — Supplementary Material 2. [file 12909_2026_8806_MOESM2_ESM.png]
