# Supplementary material for: From mannequins to humans – are manual therapy motor skills transferable? A mixed-methods study
Source: BMC Med Educ. 2026 Feb 14;26:459. doi: 10.1186/s12909-026-08806-7 (PMC13011724; doi:10.1186/s12909-026-08806-7)
Supplement: Supplementary file 3 — Supplementary Material 3. [file 12909_2026_8806_MOESM3_ESM.docx]

Supplementary File

Table 2: **Student SMT HUM Result Matrix**

● = Pass, ○ = Fail

|  |  | Test 1 | | | Test 2 | | | Test 3 | | |
| --- | --- | --- | --- | --- | --- | --- | --- | --- | --- | --- |
|  | HUM Results | Preload | Impulse | Time | Preload | Impulse | Time | Preload | Impulse | Time |
| 1 | HUM success | ○ | ● | ● | ● | ● | ● | ● | ● | ● |
| 2 | HUM success | ● | ● | ● | ● | ● | ● | ● | ● | ● |
| 3 | HUM success | ● | ● | ● | ● | ● | ● | ● | ● | ● |
| 4 | HUM success | ○ | ● | ● | ● | ● | ● | ● | ● | ● |
| 5 | HUM success | ● | ● | ● | ○ | ● | ● | ● | ● | ● |
| 6 | HUM success | ● | ● | ● | ● | ● | ● | ● | ● | ● |
| 7 | HUM success | ● | ● | ● | ○ | ● | ● | ● | ● | ● |
| 8 | HUM success | ● | ● | ● | ● | ● | ● | ● | ● | ● |
| 9 | HUM success | ● | ● | ● | ● | ● | ● | ● | ● | ● |
| 10 | HUM success | ● | ● | ● | ● | ● | ● | ● | ○ | ● |
| 11 | HUM success | ● | ● | ● | ● | ● | ● | ● | ● | ● |
| 12 | HUM success | ● | ● | ● | ● | ● | ● | ● | ● | ● |
| 13 | HUM success | ● | ● | ● | ● | ● | ● | ● | ● | ● |
| 14 | HUM success | ● | ● | ● | ● | ● | ● | ● | ● | ● |
| 15 | HUM success | ● | ● | ● | ● | ● | ● | ○ | ● | ● |
| 16 | HUM success | ● | ● | ● | ● | ○ | ● | ● | ● | ● |
| 17 | HUM success | ● | ● | ● | ● | ● | ● | ● | ● | ● |
| 18 | HUM success | ● | ○ | ● | ● | ● | ● | ● | ● | ● |
| 19 | HAM success, HUM unsuccessful | ○ | ○ | ● | ○ | ● | ● | ○ | ● | ○ |
| 20 | HUM success | ● | ● | ● | ● | ● | ● | ● | ● | ● |
| 21 | HAM success, HUM unsuccessful | ● | ● | ● | ○ | ○ | ● | ○ | ○ | ● |
| 22 | HUM success | ● | ● | ● | ● | ● | ● | ● | ● | ● |
| 23 | HAM success, HUM unsuccessful | ○ | ● | ● | ○ | ○ | ● | ○ | ○ | ● |
| 24 | HUM success | ● | ○ | ● | ● | ● | ● | ● | ● | ● |
| 25 | HUM success | ● | ● | ● | ● | ● | ● | ● | ● | ● |
| 26 | HAM success, HUM unsuccessful | ○ | ● | ● | ○ | ● | ● | ● | ● | ● |
| 27 | HUM success | ● | ● | ● | ○ | ● | ● | ● | ● | ● |
| 28 | HAM success, HUM unsuccessful | ○ | ● | ● | ○ | ● | ● | ○ | ● | ● |
| 29 | HUM success | ● | ● | ● | ● | ● | ● | ● | ● | ● |
| 30 | HUM success | ● | ● | ● | ● | ● | ● | ● | ● | ● |
| 31 | HAM success, HUM unsuccessful | ● | ● | ● | ● | ○ | ● | ● | ○ | ● |
| 32 | HAM success, HUM unsuccessful | ○ | ● | ● | ○ | ● | ● | ○ | ● | ● |
| 33 | HUM success | ● | ● | ● | ● | ● | ● | ○ | ● | ● |
| 34 | HUM success | ● | ● | ● | ● | ● | ● | ● | ● | ● |
| 35 | HAM success, HUM unsuccessful | ● | ● | ● | ○ | ● | ● | ○ | ● | ● |
| 36 | HAM success, HUM unsuccessful | ○ | ● | ● | ○ | ○ | ● | ○ | ● | ● |
| 37 | HAM success, HUM unsuccessful | ○ | ● | ● | ○ | ● | ● | ○ | ● | ● |
| 38 | HUM success | ● | ● | ● | ● | ○ | ● | ● | ● | ● |
| 39 | HAM success, HUM unsuccessful | ○ | ● | ● | ○ | ● | ● | ○ | ● | ● |
| 40 | HUM success | ● | ● | ● | ● | ● | ● | ● | ● | ● |
| 41 | HUM success | ● | ● | ● | ● | ● | ● | ● | ● | ● |
| 42 | HAM success, HUM unsuccessful | ● | ● | ● | ○ | ● | ● | ○ | ● | ● |
| 43 | HUM success | ● | ● | ● | ○ | ● | ● | ● | ● | ● |
| 44 | HUM success | ● | ● | ● | ● | ● | ● | ● | ● | ● |
| 45 | HUM success | ● | ● | ● | ● | ● | ● | ● | ● | ● |
| 46 | HUM success | ● | ● | ● | ● | ● | ● | ● | ● | ● |
| 47 | HUM success | ● | ● | ● | ○ | ● | ● | ● | ● | ● |
| 48 | HAM success, HUM unsuccessful | ○ | ○ | ● | ● | ● | ● | ○ | ● | ● |
| 49 | HUM success | ● | ● | ● | ● | ● | ● | ● | ● | ● |
| 50 | HUM success | ● | ● | ● | ● | ● | ● | ● | ● | ● |
