# Supplementary material for: From mannequins to humans – are manual therapy motor skills transferable? A mixed-methods study
Source: BMC Med Educ. 2026 Feb 14;26:459. doi: 10.1186/s12909-026-08806-7 (PMC13011724; doi:10.1186/s12909-026-08806-7)
Supplement: Supplementary file 4 — Supplementary Material 4. [file 12909_2026_8806_MOESM4_ESM.pdf]

From manikins to humans – are spinal manipulative therapy force-time characteristics delivered to manikins replicable in humans?

1. Study ID

From manikins to humans – are spinal manipulative therapy force-time characteristics delivered to manikins replicable in humans?

Information Letter

Study Title: From manikins to humans – are spinal manipulative therapy force-time characteristics delivered to mannikins replicable in humans?

Research Team:
Dr. Martha Funabashi
Canadian Memorial Chiropractic College
(647) 805-2024
mfunabashi@cmcc.ca

Co-investigators
Dr. David Starmer - Canadian Memorial Chiropractic College - dstarmer@cmcc.ca
Dr. Grand Choi - Canadian Memorial Chiropractic College - gchoi@cmcc.ca
Steve Tran - Canadian Memorial Chiropractic College – stran@cmcc.ca
Dr. Casper Nim - University of Southern Denmark – casper.nim@rsyd.dk
Nicole Smith - Canadian Memorial Chiropractic College
Kitlyn Wong - Canadian Memorial Chiropractic College
Angela Gnjatic - Canadian Memorial Chiropractic College
Dr. Samuel Howarth - Canadian Memorial Chiropractic College - showarth@cmcc.ca

Purpose of the Study
To determine if spinal manipulative therapy (SMT) force-time characteristics (i.e., impulse peak force, time to peak and loading rate) delivered to manikins are replicable in humans.

Procedures Involved in this Study and Time Commitment
You will be asked to come to the FSTT® Laboratory at the Canadian Memorial Chiropractic College for data collection. You will be required for 1 unique session, which should take approximately 30-40 minutes in total.

This session will include:

Procedures
• Explanation of the study and the technique that you will be asked to perform on a manikin and/or receive.
• Choosing to participate as a "provider" (i.e., applying the SMT), and/or as a human to receive the SMT.

If you agree to participate as a "provider":
• You will be asked to perform posterior-to-anterior thoracic SMTs to a manikin on the FSTT® in the following steps:

o Define the preload force magnitude you judge to be appropriate
o Apply 5 SMTs for each of the following force-time characteristics:
▪ The pre-defined preload (±50N), 200N (±75N) impulse with a time to peak <200ms using any hand contact you prefer

If you agree to participate as a volunteer who receives the SMT:
• You will be asked to lie down in prone of the FSTT®
• A "provider" participant will apply an initial force to define the preload force magnitude that is unique to you
• Once preload is defined, you will then receive up to 3 posterior-to-anterior thoracic SMTs with a 200N (±75N) impulse force.

Potential Risks and Associated Safeguards
• Regardless if you participate as a "provider" and/or as receiving the SMTs, you may experience discomfort during the SMT application. You may discontinue at any time without penalty, especially if you are experiencing discomfort. If you feel uncomfortable at any point throughout the testing protocol, let the investigators know and they will discontinue the test.
• Although unlikely, muscle and ligament strain are potential risks with thoracic manipulation. Typically, this includes Grade 1 strains, which will likely resolve itself over the course of a few days with rest and minor care. Similarly, although unlikely, rib fractures are also potential risks during thoracic spinal manipulation. While the investigators have taken reasonable precautions to minimize risk, in the unlikely event of an injury or emergency during testing, you will be immediately referred for evaluation to the chiropractic clinic located on campus, and standard emergency procedures will be followed should they be required.

Changing Your Mind about Participation
You may withdraw from the study at any time without any penalty. To do so, indicate this to the investigators by saying, "

Potential Benefits of Participation
Information obtained from this research may aid in the understanding the transferability of manual therapy motor skills acquired from practicing on manikins to humans. This, in turn, can inform training of chiropractic students. You may withdraw from participating in the study at any time without any consequences.

Confidentiality
Each participant will be assigned a unique identification code consisting of a three numbers. Only the principal investigator of this study will have access to this code and the data related to this study. Any identifiable information will be destroyed once data collection is complete. All unidentifiable data will be stored indefinitely on computer hard drives (password protected). Only the study investigators involved in the data analysis (MF, CN and SH) will have access to the data. Data will remain anonymous for long term storage and may be used in future research (secondary analysis). All data will be analyzed in a combined manner and no individual data will be analyzed or presented at any time.

Concerns about Participation
We would like to assure you that this study has been reviewed by, and received ethics clearance through, CMCC's Research Ethics Board (REB). However, the final decision about participation is yours. In the event you have any comments or concerns resulting from your participation in this study, please contact Mr. Mark Fillery at (647) 805-2022.

Questions about the Study
If you have any further questions or want any other information about this study, please feel free to ask the investigators. If you have additional questions at a later date, please contact Dr. Martha Funabashi at (647) 805-2024 or by e-mail at mfunabashi@cmcc.ca.

Sincerely Yours,
Dr. Martha Funabashi
(647) 805-2024
mfunabashi@cmcc.ca

* 2. Do you consent to having your unidentifiable data to be used in the future for secondary analyses?

○ Yes

○ No

* 3. Do you agree to participate in this study?

○ Yes

○ No

4. Once this study is completed, would you like to receive of copy of the results summary?

○ Yes

○ No

From manikins to humans – are spinal manipulative therapy force-time characteristics delivered to manikins replicable in humans?

5. Please enter the email address you would like the results summary to be sent to.

[ ]

From manikins to humans – are spinal manipulative therapy force-time characteristics delivered to manikins replicable in humans?

Demographic and Anthropometric Information

* 6. Full name:

[ ]

* 7. Sex

- ( ) Female
- ( ) Male

* 8. Gender

- [ ] Gender Fluid
- [ ] Man
- [ ] Woman
- [ ] Trans man
- [ ] Trans woman
- [ ] Two spirit
- [ ] Nonbinary
- [ ] I do not identify with any
- [ ] Prefer not to answer
- [ ] Other

9. If other, please specify

[ ]

From manikins to humans – are spinal manipulative therapy force-time characteristics delivered to manikins replicable in humans?

* 10. How old are you? (in years)

[ ]

* 11. What is your height? (in inches)

[ ]

* 12. What is your weight? (in lbs)

* 13. Are you currently receiving any treatment for your mid back (thoracic region)?

- Yes
- No

* 14. What year of the chiropractic program are you currently in?

- 1st year
- 2nd year
- 3rd year
- 4th year

* 15. You agree to participate as a:

- Provider
- Receiving the SMT
- Both

From manikins to humans – are spinal manipulative therapy force-time characteristics delivered to manikins replicable in humans?

Please hand the tablet to the research assistant to proceed to the first part of the study

From manikins to humans – are spinal manipulative therapy force-time characteristics delivered to manikins replicable in humans?

HAM Data Collection Tracking

* 16. HAM Preload

* 17. HAM Trial 1

- Pass
- Not pass - Preload
- Not pass - Impulse
- Not pass - Time to peak

* 18. HAM Trial 2

- [ ] Pass
- [ ] Not pass - Preload
- [ ] Not pass - Impulse
- [ ] Not pass - Time to peak

* 19. HAM Trial 3

- [ ] Pass
- [ ] Not pass - Preload
- [ ] Not pass - Impulse
- [ ] Not pass - Time to peak

* 20. HAM Trial 4

- [ ] Pass
- [ ] Not pass - Preload
- [ ] Not pass - Impulse
- [ ] Not pass - Time to peak

* 21. HAM Trial 5

- [ ] Pass
- [ ] Not pass - Preload
- [ ] Not pass - Impulse
- [ ] Not pass - Time to peak

* 22. Pass to HUM

- ○ Yes
- ○ No

From manikins to humans – are spinal manipulative therapy force-time characteristics delivered to manikins replicable in humans?

Survey II - Between Manikin and Human

* 23. Have you practiced any technique with this person before?

- ○ Yes
- ○ No

From manikins to humans – are spinal manipulative therapy force-time characteristics delivered to manikins replicable in humans?

Please hand the tablet to the research assistant to proceed to the next phase of the study

From manikins to humans – are spinal manipulative therapy force-time characteristics delivered to manikins replicable in humans?

HUM Data Collection Tracking

* 24. HUM Preload

[ ]

* 25. HUM Trial

- [ ] 1
- [ ] 2
- [ ] 3

From manikins to humans – are spinal manipulative therapy force-time characteristics delivered to manikins replicable in humans?

Survey III - After completion of SMT data collection

* 26. Please indicate how anxious you were with applying SMT with the specific force-time characteristics (pre-determine pre-load (±50N), followed by an impulse of 200N (±75N) with a thrust duration of <200ms) using the following visual diagram:

0 - No anxiety | 100 - Maximum anxiety

* 27. Do you think you were able to replicate the SMT force-time characteristics (pre-determined pre-load (±50N), followed by an impulse of 200N (±75N) with a thrust duration of <200ms) to this specific person?

- ○ Yes
- ○ Partially
- ○ No

From manikins to humans – are spinal manipulative therapy force-time characteristics delivered to manikins replicable in humans?

* 28. Please describe and explain why you think that

From manikins to humans – are spinal manipulative therapy force-time characteristics delivered to manikins replicable in humans?

* 29. Do you think a SMT an impulse of 200N (±75N) with a thrust duration of <200ms is appropriate for this person?

- ○ Yes
- ○ No

From manikins to humans – are spinal manipulative therapy force-time characteristics delivered to manikins replicable in humans?

* 30. Please explain

From manikins to humans – are spinal manipulative therapy force-time characteristics delivered to manikins replicable in humans?

* 31. Do you think the hand contact you used was appropriate for this specific person?

- ○ Yes
- ○ No

From manikins to humans – are spinal manipulative therapy force-time characteristics delivered to manikins replicable in humans?

* 32. Please explain

* 33. What hand contact would you have used instead and why?

From manikins to humans – are spinal manipulative therapy force-time characteristics delivered to manikins replicable in humans?

* 34. How do you think the person was different from the manikin?

From manikins to humans – are spinal manipulative therapy force-time characteristics delivered to manikins replicable in humans?

* 35. Did you hear a cavitation during any of the SMT trials in this person?

- Yes
- No

From manikins to humans – are spinal manipulative therapy force-time characteristics delivered to manikins replicable in humans?

* 36. Did the cavitation influence how you applied the following SMT trials?

- Yes
- No

From manikins to humans – are spinal manipulative therapy force-time characteristics delivered to manikins replicable in humans?

* 37. Please describe how did the cavitation influence your SMT application?

From manikins to humans – are spinal manipulative therapy force-time characteristics delivered to manikins replicable in humans?

Thank you very much!

Please return the tablet to the investigator.

Thank you!
